# The Impact of and Government Planning and Responses to Pandemics for People with Disability: A Rapid Review

**DOI:** 10.3390/ijerph18126505

**Published:** 2021-06-16

**Authors:** Adyya Gupta, Anne Kavanagh, George Disney

**Affiliations:** 1 Global Obesity Centre, Institute for Health Transformation, Deakin University, Geelong, VIC 3220, Australia; 2 Centre for Health Equity, Disability and Health Unit, Melbourne School of Population and Global Health, University of Melbourne, Melbourne, VIC 3010, Australia; a.kavanagh@unimelb.edu.au (A.K.); George.disney@unimelb.edu.au (G.D.)

**Keywords:** disability, pandemic, outbreak, response, emergency preparedness, risk of infection

## Abstract

*Objective*—To collate evidence on (1) the risk of infection for people with disability during infectious disease outbreaks and/or pandemics and (2) government responses and pandemic plans for people with disability. *Methods*—Through two rapid reviews, relevant peer-reviewed studies and grey literature published from 2002 onwards in the English language were identified. Data were synthesised narratively. *Results*—Aim 1: Of the 680 studies, two studies were included in the review. No grey literature was eligible for inclusion. The evidence regarding risk was inconclusive. Aim 2: Of the 50 studies, three peer-review studies, along with four government reports were included. The literature largely reported on measures being taken to maximise the prevention of transmission of COVID-19 for the general population, with only a few programs including people with disability. *Conclusion*—Overall, there is inconclusive evidence on the risk of infection for people with disability during infectious disease outbreaks and/or pandemics and the government preparedness and planning for disease outbreaks and/or pandemics largely exclude people with disability. From a population health perspective, during disease outbreaks and pandemics, including the COVID-19 pandemic, along with the general population, it is important for governments to include people with disability in their pandemic planning and response.

## 1. Introduction

According to the International Classification of Functioning, Disability and Health, disability is defined as an “umbrella term for impairments, activity limitations, and participation restrictions; it denotes the negative aspects of the interaction between an individual (with a health condition) and that individual’s contextual factors (environmental and personal)” [1]. More than a billion people (15% of the world’s population) live with some form of disability worldwide and around 785 million (15.6%) people with disability are 15 years and older [2]. Over 4.4 million (1 in 5) people in Australia live with some form of disability [3]. Nearly half (44.5%) of the population with a disability are aged 65 years and over [3]. Furthermore, people with disability experience significant inequalities in health [4].

Epidemics and pandemics pose a serious threat to the health of children and adults with disability, their families, carers and all those that work (both directly and/or indirectly) with people with disability. The recent global pandemic, COVID-19 has shown itself to have profound impacts on all groups of society worldwide, and specifically places people with disability at an increased risk of harm. This is because people with disability are especially vulnerable to the physical, mental and social effects of the pandemic. People with disability are at an increased risk of exposure to SARS-CoV-2 (e.g., through contact with multiple support workers or potentially poor hygiene practices) and many have high risks of complications and death if infected because of underlying health problems [4,5,6].

Evidence from the aftermath of previous emergencies, such as storms and hurricanes, shows that people with disability are often disproportionately harmed, as emergency preparedness plans are often not tailored to their needs [7]. Despite the potential risks to people with disability in pandemics and evidence that they fare poorly in emergencies, people with disability were not prioritised by governments around the world in responding to COVID-19.

To assist governments and the disability sector to understand the risk to people with disability and respond to those risks, we conducted two rapid reviews. The first aimed to collate evidence on the risk of infection for people with disability during infectious disease outbreaks and/or pandemics. This was done to understand previous evidence on the extent to which people with disability were infected, the settings in which the infection was acquired (exposure site, e.g., private home, residential care setting or health facility) and which sub-groups within the population of people with disability were at greater risk of infection and poor health outcomes. The second rapid review aimed to gather government responses to previous infectious disease outbreaks and pandemic plans for people with disability. This will contribute to the pandemic preparedness and public health response aimed at reducing the risks of COVID-19 posed to populations with disability.

## 2. Materials and Methods

A review protocol was developed a priori using the PROSPERO International prospective register of systematic reviews form. For the reporting of this rapid review, the PRISMA (Preferred Reporting Items for Systematic Reviews and Meta-Analyses) guideline for systematic reviews was followed to streamline the rapid review process [8].

### 2.1. Search Strategy

Using a range of keywords, the search strategy was developed for PubMed. Search terms categorised under three broad hedge headings (population, setting/policy and pandemic) were combined using the Boolean operator “AND”.

For aim 1, the search terms included under the hedge heading population were (Disabilit* OR Disable* OR Vulnerable Population* OR impairment OR psych* OR mental health). The search terms under hedge heading setting/policy were (social care OR home or domiciliary OR community OR outreach OR care OR “healthcare” OR resident* OR “psychiatric ward”). The search terms under hedge heading pandemic were (Epidemic* OR Pandemic* OR outbreak* OR Disease Outbreaks OR Influenza OR emergency OR cluster* OR respiratory* OR coronavirus OR corona OR COVID-19 OR Ebola OR SARS OR H1N1 OR Zika OR disease OR virus OR “risk of infection”) (Table S1: Search strategy).

For aim 2, the search terms included under the hedge heading population were (Disabilit* OR Disable* OR Vulnerable Population* OR impairment OR psych* OR mental health). The search terms under hedge heading setting/policy were (polic* OR plan* OR resource* OR government* OR respons* OR prepar*). The search terms under hedge heading pandemic were (Epidemic* OR Pandemic* OR outbreak* OR Disease Outbreaks OR Influenza OR emergency OR cluster* OR respiratory* OR coronavirus OR corona OR COVID-19 OR Ebola OR SARS OR H1N1 OR Zika OR disease OR virus OR “risk of infection”) (Table S1: Search strategy).

The search for the relevant literature was also augmented by searching through the reference lists of the included articles and the scientific literature known to the authors. A grey literature search was also conducted for both aims of this study to search the following government websites of selected high-income countries: Australian Government (.gov.au), Australian organisations (.org.au), the United States (U.S.) of America’s Department of Health (.hhs.gov), United Kingdom Government (.gov.uk) and Canadian Government (.gc.ca). We only included selected high-income countries to search for grey literature because we were keen to gather evidence from countries that were comparable to Australia to help inform our study’s recommendations. It was predicted that the Google search would return a large volume of websites and therefore the screening was limited to the first 50 uniform resource locators (URLs) from each website host and, depending on the relevancy, the next 50 URLs were searched thereafter.

### 2.2. Inclusion and Exclusion Criteria

All peer-reviewed studies published from 2002 to October 2020 in the English language were included. To address the two aims of this review, a study selection criterion was developed using a modified PICOS (population, intervention/exposure, context, outcome and study type) format. This is described as follows.

Population: People with disability. We also included people with long-term mental health conditions (psychosocial disability), residents in psychiatric wards or the community. We included individuals with mental health conditions because we were interested in identifying people with long-term psychosocial disability residing in psychiatric wards or the community.

Intervention/Exposure: Infectious disease outbreaks and/or pandemics. According to the World Health Organisation (WHO), infectious disease outbreaks are defined as the occurrence of a disease usually caused by an infection, transmitted through person-to-person contact, animal-to-person contact, or from the environment or other media, in excess of normal expectancy [9]. A pandemic is defined as a worldwide spread of a new disease [10]. For the purpose of this rapid review, we followed the WHO definitions of infectious disease outbreaks and pandemics.

Context:

For aim 1: To document the extent to which people with disability were infected, the settings in which the infection was acquired (exposure site) and which sub-groups within the population of people with disability were at greater risk of infection and poor health outcomes.

For aim 2: To document the government responses to previously experienced infectious disease outbreaks and pandemic plans for people with disability.

Outcome:

For aim 1: Primary outcome of interest is the risk of infection from infectious disease during disease outbreaks and/or pandemics.

Secondary outcomes of interest were (1) socio-demographic characteristics of people with disability most at risk of infection from infectious disease during disease outbreaks from pandemics and (2) the exposure site of the infection (the setting in which the people with disability who are infected live or work, e.g., private home, residential care setting or health facility)

For aim 2: The primary outcome of interest was government responses to previously experienced infectious outbreaks and pandemic plans for people with disability.

Study type: For both the aims of this rapid review, all study designs including randomised controlled trials (RCT), cohort studies, case–control studies, cross-sectional studies and study types, such as open letters, essays and commentaries, were considered eligible for inclusion. Letters to the editor, conference abstracts and reviews were excluded. No study was excluded based on race, culture, ethnicity or geographical location.

### 2.3. Quality Assessments

As the premise of this rapid review was to determine the extent, range and nature of the research available, making it a more suitable approach than a systematic review for this research question, we did not undertake any risk of bias assessment [11]. Furthermore, a quality assessment of the selected studies was not conducted, as our rapid review did not aim to synthesise evidence according to methodological quality [11]. This decision was made in consultation with the team of authors.

### 2.4. Study Screening Process

All relevant studies identified through the search were imported into the Covidence database. Covidence is used for managing and streamlining systematic review processes. Following the removal of duplicates, title and abstract screening of articles were conducted (by A.G. and G.D.) to identify articles meeting the inclusion and exclusion criteria using a screening checklist. Two independent reviewers screened the title/abstracts of the articles identified and resolved any discrepancies. All the screening process for this review was conducted using the PRISMA guidelines (Table S2: PRISMA checklist) [8]. To identify the final list of eligible studies, the screening checklist was reapplied by A.G. and G.D., and the studies not meeting the inclusion criteria were excluded from the review.

### 2.5. Data Synthesis

A formal data extraction matrix table was not developed as only a limited number of studies were included in this rapid review. However, all the relevant data were extracted from all included articles and were described narratively. All discrepancies in the interpretation of the data extracted were resolved through discussions between the co-authors. The final data from the included studies were synthesised narratively and presented separately for aims 1 and 2 for clarity and appropriateness. Studies were thoroughly read to identify any mention of vulnerable groups or groups at risk. If the included studies identified and/or reported on the vulnerable groups or groups at risk, they were categorised and synthesised narratively.

## 3. Results

### 3.1. Aim 1

**Figure 1:**
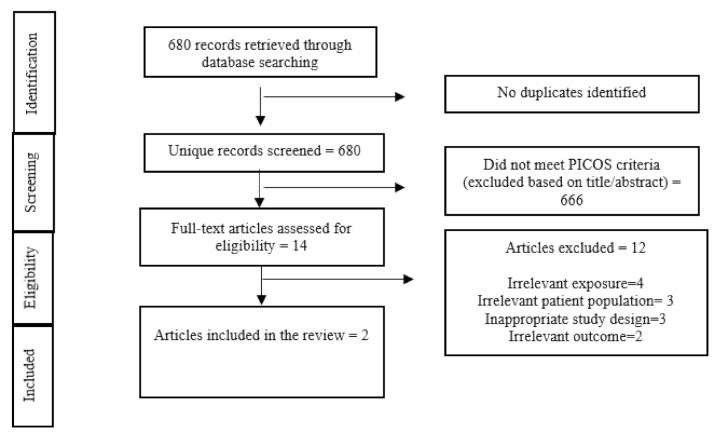
Flow diagram of included studies.

Of the 680 studies identified through our systematic search (Figure 1 Flow diagram of included studies), fourteen studies underwent a full-text screening. Of these, only two studies [12,13] fulfilled the eligibility criteria and were included in the review. No relevant grey literature met the eligibility criteria for inclusion in this review.

#### Overview of Included Studies

The two studies included in the review were conducted in France [12] and Israel [13]. The study by Chevance et al. [12] is a literature review that summarises the gaps in the mental healthcare system gathered from experiences of a previous famine in psychiatric hospitals during the Second World War in France. To help the mental healthcare system cope with the current SARS-CoV-2 epidemic in France, the review proposed the need for a reorganisation of the system. The authors’ argued that pre-existing medical comorbidities, cognitive and behavioural disorders, psychosocial vulnerability (including socio-economic living conditions) and older age made people with diagnoses of mental disorders at risk of being infected during a pandemic. Recommendations for a reorganisation of the mental healthcare system in response to the SARS-CoV-2 epidemic included establishing a COVID unit within the psychiatry ward, creation of an integrated COVID and medical/psychiatric system within the medical units, launching a telephone hotline. No information on the review methodology was provided by the authors.

The other study by Ghanaiem et al. [13], published a decade earlier, was a case study, that described the risk of developing severe and life-threatening respiratory tract infection due to adenovirus type 7 outbreak in a paediatric residential facility. The paediatric residential facility accommodated 62 children with severe physical and/or cognitive disabilities. Eight children (median age of 22.5 months (age range: 9 months to 5 years)), that shared rooms (particularly playroom) at the paediatric residential facility reported respiratory distress with hypoxemia. No children had any prior immunodeficiency or chronic lung disease, and none were ventilator dependent. The spread of infection was through droplets of one of the twelve staff members who were in close contact with the children while showing early symptoms of fever, conjunctivitis and sore throat.

### 3.2. Aim 2

**Figure 2:**
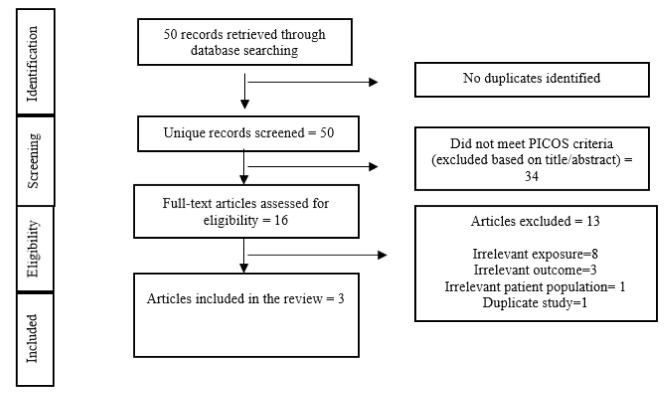
Flow diagram of included studies.

Of the 50 studies identified through our systematic search process (Figure 2 Flow diagram of included studies), 16 studies underwent a full-text screening. Of these only three studies (a commentary [14], an open letter [15] and an essay [16]) met the criteria for inclusion and were included in the review. Furthermore, a grey literature search was conducted that led to the identification of 20 relevant reports. Of these four reports met the eligibility criteria and were included in the review.

#### Overview of Included Studies

Two [14,15] (a commentary and an open letter) of the three peer-reviewed articles discussed the need to include people with disability in the government’s public health response to the COVID-19 pandemic. The third peer-reviewed article was an essay [16] that identified the potential needs of people with disability and discussed strategies to plan a more inclusive response to pandemic influenza. Of the four grey literature documents included in this review, three documents [17,18,19] discussed the government’s preparedness plans to assist people with disability and one document was a guide [20] to assist people with disability to manage their own emergency preparedness plan, all for the ongoing COVID-19 pandemic. Two of the four grey literature documents were published in Australia [17,20] and one each by United Kingdom aid (UKaid) [18] and the WHO [19].

These grey literature documents largely illustrated examples of current programs in place to facilitate community preparedness and maximise the prevention of transmission of COVID-19 for the general population, with only a few programs including some information for people with disability. For example, one document mentioned that King’s College London launched a population-wide C-19 COVID Symptom Study app (developed by the health science company ZOE) to identify high-risk areas and population groups that need immediate attention [21]. This app is used by people living in the United Kingdom (UK) to regularly report on their health, their need for regular help and having a health problem that requires staying at home and regularly using a stick, walking frame or wheelchair to get about. It is endorsed by the Welsh Government, NHS Wales, the Scottish Government and NHS Scotland. The U.K. has also established British Sign Language resources for people with hearing loss or in easy English for those with intellectual disability. These groups may lack necessary public health information about the virus due to public restrictions, such as physical isolation, stay-at-home orders and reduced social contacts. The Alzheimer’s Association, also in U.K., has developed guidance for caregivers of people with dementia to explain hygienic behaviours, such as handwashing, and suggests how to keep the person healthy and safe [22]. Furthermore, in the U.K., the National Health Services (NHS) has developed guidelines on how to include people with mental health conditions, learning disabilities and autism within the COVID-19 response [23]. The documents published in Australia stated that the Australian Government is in regular consultation with state and territory governments and health organisations to update their emergency preparedness plans to support the disability community [24].

## 4. Discussion

The aims of this rapid review were to review evidence on the (1) risk of infection for people with disability during infectious disease outbreaks and/or pandemics and (2) government preparedness and planning for infectious disease outbreaks and/or pandemics. First, due to the lack of any robust empirical data, there is inconclusive evidence on the risk of infection for people with disability during infectious disease outbreaks and/or pandemics, despite increased mortality rates for people with disability due to COVID-19 [25]. However, recent data from England and the United States is concerning. So far, in England, there have been 705 deaths among people with intellectual and developmental disabilities (IDD) due to COVID-19 [26]. There is evidence that people with learning disability are twice as likely to contract COVID-19 infection and three and four times more likely to die of COVID-19 [27]. Data from the United States shows there are a greater concentration of COVID-19 cases in younger people and a higher rate of pre-existing conditions related to COVID-19 disease severity and mortality among people with IDD than among people without IDD [28]. Further data is required to identify the level of risk of infection for people with a disability and if there is, then gathering data on the exposure site and the nature of chains of transmission (including mode of transmission (airborne (with variations) vs. contact (direct, indirect, surface variations)) that lead to infections among the whole population of people with disability is essential. Second, the evidence on the government preparedness and planning for infectious disease outbreaks and/or pandemics was also limited in scope. However, from within the limited evidence, it is clear that the focus of government preparedness and planning for infectious disease outbreaks and/or pandemics is largely for the general population with little or no plan for people with disability.

Moving forward, we believe it is critical to collect individual-level disability data and other relevant risk factors (e.g., type of home or accommodation, number of people living in a household) and health outcomes related to COVID-19, such as infection, illness, hospitalisations and deaths. This will help us understand the specific source of infection among people with disability, their families and paid support workers and whether people with disability are at a higher risk of infection, illness and death than the population of people without disabilities. Individual-level data should include variables for disability-related factors that increase the risk for exposure (e.g., exposure to multiple support workers), complications and death from infectious disease outbreaks and/or pandemic influenza.

Evidence suggests that a lack of individual-level data about the characteristics of residents living in long-term care facilities is a barrier to planning and preparing for the pandemics like the COVID-19 pandemic [29,30]. This has meant that mathematical modelling that informed government planning for the pandemic did not account for people with disability in their estimations.

In responding to COVID-19, the authors believe it is imperative for governments around the world to prioritise testing, contact tracing and monitoring (the spread of infection, if any) among people receiving and providing care services to people with disability. There is evidence that through rapid testing and assessments, the general population, including people with disability, are encouraged to get tested for early identification of cases and treatment. Several actions are undertaken in many countries when a laboratory confirmation of an infection is received. These include the isolation of confirmed cases, offer assistance to help maintain good hygiene and contact tracing. Contact tracing is another critical measure that is been extensively undertaken in many countries to reduce community transmission of the disease. Engaging with organisations, such as Disabled Peoples Organisations (DPOs) and Disability Representative Organisations (DROs), is critical for ensuring an inclusive emergency preparedness plan in the COVID-19 pandemic [31].

Second, the evidence suggests that designing clear and tailored communication will help make public health messaging more accessible for people with disability. This includes providing resources to people with disability, their family and caregivers on the risk associated with the pandemic; how to protect yourself; how to manage health and hygiene during public health emergency; knowing what to do if you or someone who supports you experience symptoms; whom to contact for more information, guidance and support.

Third, the authors would like to emphasise that countries need to publish epidemiological evidence on the progression of the COVID-19 pandemic among people with disability. Countries may be able to draw on learnings from other countries that have (or have not) been successful in mitigating the harms of outbreaks for people with disability.

Last, from previous experience during disease outbreaks and pandemics, it is clear that monitoring and evaluating the ongoing strategies are critical to ensuring that future public health responses aimed at protecting people with disability are effective and feasible. This could help inform future pandemic planning, preparedness and the responses of countries globally to better prepare for and reduce the potential risks posed to populations with disabilities.

### Strengths and Limitations

This rapid review presents the most updated evidence on the extent of the risk of infection among people with disability during pandemics and the government responses to infectious disease outbreaks and pandemic plans for people with disability. It must be noted that there is substantial grey literature globally illustrating pandemic plans in general; however, only a few include specific plans targeted at people with disability. There are several strengths to this rapid review. First, a robust search strategy was developed and adapted to a large database (PubMed) to ensure all relevant studies (both academic and grey literature) were identified. Second, we developed a well-defined study selection criterion to ensure our review process was rigorous and robust. Third, findings were summarised narratively to offer a clear presentation of the findings. However, our review also has some limitations. Our search strategy did not include terms such as “mental disorders” or to identify people with IDD, schizophrenia, dementia, multiple sclerosis or somatic health-related search terms. This may have led to the exclusion of potentially relevant papers. By applying an English language limit in our search, it is possible that we may have missed some potentially relevant studies. As we searched government websites of selected high-income countries and publications published in the English language only, this may have resulted in the exclusion of potentially relevant government documents from other countries that were published in other languages. Furthermore, as evidence on COVID-19 is rapidly emerging, it may be that relevant evidence may have emerged after completing the search process for this rapid review. It is important to note that we only found one case study and thus the quality of the evidence on the risk of infection is very weak. Our ability to draw inferences from previously successful pandemic plans was limited, as the literature primarily focussed on the current COVID-19 pandemic.

## 5. Conclusions

Overall, due to limited evidence and in light of the limitations of the review, there is an evidence gap regarding whether people with disability are at risk of infection, the extent and the settings in which the infection can be acquired (exposure site), and whether or not there are any sub-groups within the population of people with disability that are at greater risk of infection and poor health outcomes during infectious disease outbreaks and/or pandemics. Further, there is limited consideration of the needs of people with disability in government preparedness and planning for disease outbreaks and/or pandemics. There is a need for better data collection, such that it should include variables relevant for monitoring groups at high risk such as the people with disabilities. This type of inclusive data collection will help with better disaggregation of data on the pandemic situation and the response for these groups. Beyond the threat presented to the general population, it is evident that a pandemic, such as the COVID-19 pandemic, poses a substantial risk to people with disability. It is imperative to have contingency plans for people living in long-term care (including people with disability) and their support staff or carers in the event of a pandemic. Including disability stakeholders (including people with disability) in all stages of planning, implementation and monitoring for managing infectious disease outbreaks and/or pandemics is essential to ensure that policies adequately address the needs of these groups to minimise the risk of harm.

## Data Availability

The authors declare that the data of this research is available from the corresponding author on request.

## Supplementary Materials

The following are available online at https://www.mdpi.com/article/10.3390/ijerph18126505/s1, Table S1: Search strategy; Table S2: PRISMA checklist.

## References

[1] World Health Organization (2001). International Classification of Functioning, Disability and Health.

[2] World Health Organization (2011). World Report on Disability.

[3] Australian Bureau of Statistics (2019). 4430.0—Disability, Ageing and Carers.

[4] Bickenbach J.E.C.A., Sabariego C. (2016). Disability and public health. Int. J. Environ. Res. Public Health.

[5] Emerson E., Hatton C. (2014). Health Inequalities and People with Intellectual Disabilities.

[6] Krahn G.L., Walker D.K., Correa-De-Araujo R. (2015). Persons with disabilities as an unrecognized health disparity population. Am J. Public Health.

[7] US House of Representatives (2006). A Failure of Initiative: Final Report of the Select Bipartisan Committee to Investigate the Preparation for and Response to Hurricane Katrina.

[8] Moher D., Liberati A., Tetzlaff J., Altman D.G. (2009). Preferred reporting items for systematic reviews and meta-analyses: The PRISMA statement. Ann. Intern. Med..

[9] World Health Organisation (2009). Disease Outbreaks.

[10] World Health Organisation (2010). What Is a Pandemic?. https://www.who.int/csr/disease/swineflu/frequently_asked_questions/pandemic/en/.

[11] Arksey H., O’Malley L. (2005). Scoping studies: Towards a methodological framework. Int. J. Soc. Res. Methodol..

[12] Chevance A., Gourion D., Hoertel N., Llorca P.M., Thomas P., Bocher R., Moro M.R., Laprévote V., Benyamina A., Fossati P. (2020). Ensuring mental health care during the SARS-CoV-2 epidemic in France: A narrative review. Encephale.

[13] Ghanaiem H., Averbuch D., Koplewitz B.Z., Yatsiv I., Braun J., Dehtyar N., Wolf D.G., Mandelboim M., Engelhard D. (2011). An outbreak of adenovirus type 7 in a residential facility for severely disabled children. Pediatr. Infect. Dis. J..

[14] Boyle C.A., Fox M.H., Havercamp S.M., Zubler J. (2020). The public health response to the COVID-19 pandemic for people with disabilities. Disabil. Health J..

[15] Kuper H., Banks L.M., Bright T., Davey C., Shakespeare T. (2020). Disability-inclusive COVID-19 response: What it is, why it is important and what we can learn from the United Kingdom’s response. Wellcome Open Res..

[16] Campbell V.A., Gilyard J.A., Sinclair L., Sternberg T., Kailes J.I. (2009). Preparing for and responding to pandemic influenza: Implications for people with disabilities. Am. J. Public Health.

[17] Australian Government (2020). Management and Operational Plan for People with Disability.

[18] Meaney-Davis J., Lee H., Corby N. (2020). The Impacts of COVID-19 on People with Disabilities.

[19] World Health Organization (2020). Preventing and Managing COVID-19 across Long-Term Care Services: Policy Brief.

[20] Villeneuve M., Moss M., Abson L., Buchanan R. (2020). Person-Centred Emergency Preparedness Planning for COVID-19.

[21] Kings College London C-19 COVID Symptom Tracker 2020. https://covid.joinzoe.com/.

[22] Alzheimers Association Coronavirus (COVID-19): Tips for Dementia Caregivers 2020. https://www.alz.org/help-support/caregiving/coronavirus-(covid-19)-tips-for-dementia-care.

[23] NHS Responding to COVID-19: Mental Health, Learning Disability and Autism NHS 2020. https://www.england.nhs.uk/coronavirus/wp-content/uploads/sites/52/2020/03/COVID19_Mental-Health-Learning-Disabilities-and-Autism-cell-update-number-2_25-March.pdf.

[24] Disability Royal Commission (2020). Disability Royal Commission and COVID-19. https://disability.royalcommission.gov.au/news-and-media/coronavirus-covid-19-update.

[25] World Health Organization Disability and Health 2018. https://www.who.int/news-room/fact-sheets/detail/disability-and-health.

[26] (2020). England. N. Covid-19 Deaths of Patients with a Learning Disability Notified to Learning from Death Reviews (LeDeR). https://www.england.nhs.uk/publication/covid-19-deaths-of-patients-with-a-learning-disability-notified-to-leder/.

[27] Public Health England (2020). Disparities in the Risk and Outcomes from COVID-19. https://assets.publishing.service.gov.uk/government/uploads/system/uploads/attachment_data/file/908434/Disparities_in_the_risk_and_outcomes_of_COVID_August_2020_update.pdf.

[28] Turk M., Landes S.D., Formica M.K., Goss K.D. (2020). Intellectual and developmental disability and COVID-19 case-fatality trends: TriNetX analysis. Disabil. Health J..

[29] Burton J., Goodman C., Quinn T. (2020). The Invisibility of the UK Care Home Population: UK Care Homes and a Minimum Dataset. https://ltccovid.org/2020/05/14/the-invisibility-of-the-uk-care-home-population-uk-care-homes-and-a-minimum-dataset/.

[30] Romero-Ortuño1 R., Kennelly S. (2020). COVID-19 Deaths in Irish Nursing Homes: Exploring Variation and association with the Adherence to National Regulatory Quality Standards. https://ltccovid.org/2020/06/01/covid-19-deaths-in-irish-nursing-homes-exploring-variation-and-association-with-the-adherence-to-national-regulatory-quality-standards/.

[31] Disabled People’s Organisations Australia (2020). An Open Letter to the National Cabinet: Immediate Actions Required for Australians with Disability in Response to Coronavirus (COVID19).

